# Effects of Deworming on Malnourished Preschool Children in India: An Open-Labelled, Cluster-Randomized Trial

**DOI:** 10.1371/journal.pntd.0000223

**Published:** 2008-04-16

**Authors:** Shally Awasthi, Richard Peto, Vinod K. Pande, Robert H. Fletcher, Simon Read, Donald A. P. Bundy

**Affiliations:** 1 Department of Paediatrics, King George's Medical University, Lucknow, India; 2 Clinical Trial Service Unit, University of Oxford, Oxford, United Kingdom; 3 Harvard Medical School, Harvard University, Boston, Massachusetts, United States of America; 4 Human Development Division, The World Bank, Washington, D.C., United States of America; The George Washington University, United States of America

## Abstract

**Background:**

More than a third of the world's children are infected with intestinal nematodes. Current control approaches emphasise treatment of school age children, and there is a lack of information on the effects of deworming preschool children.

**Methodology:**

We studied the effects on the heights and weights of 3,935 children, initially 1 to 5 years of age, of five rounds of anthelmintic treatment (400 mg albendazole) administered every 6 months over 2 years. The children lived in 50 areas, each defined by precise government boundaries as urban slums, in Lucknow, North India. All children were offered vitamin A every 6 months, and children in 25 randomly assigned slum areas also received 6-monthly albendazole. Treatments were delivered by the State Integrated Child Development Scheme (ICDS), and height and weight were monitored at baseline and every 6 months for 24 months (trial registration number NCT00396500). p Value calculations are based only on the 50 area-specific mean values, as randomization was by area.

**Findings:**

The ICDS infrastructure proved able to deliver the interventions. 95% (3,712/3,912) of those alive at the end of the study had received all five interventions and had been measured during all four follow-up surveys, and 99% (3,855/3,912) were measured at the last of these surveys. At this final follow up, the albendazole-treated arm exhibited a similar height gain but a 35 (SE 5) % greater weight gain, equivalent to an extra 1 (SE 0.15) kg over 2 years (99% CI 0.6–1.4 kg, p = 10^−11^).

**Conclusions:**

In such urban slums in the 1990s, five 6-monthly rounds of single dose anthelmintic treatment of malnourished, poor children initially aged 1–5 years results in substantial weight gain. The ICDS system could provide a sustainable, inexpensive approach to the delivery of anthelmintics or micronutrient supplements to such populations. As, however, we do not know the control parasite burden, these results are difficult to generalize.

**Trial Registration:**

ClinicalTrials.gov NCT00396500

## Author Summary

About one-third of children in poor communities globally are infected with intestinal worms. Treatment is effective and safe, and involves taking a pill once or twice a year. Most deworming programs are aimed at children of school age because most infections occur in this age group and schoolchildren are easy to reach and treat in schools. But preschool children are also infected and, in North India, the State Integrated Child Development Scheme (ICDS) provides a system of preschools and teachers that could potentially deliver treatment to younger children. To see whether deworming would be feasible and beneficial for preschool children, we studied its effects on the growth of 4,000 children initially aged 1 to 5 years in the urban slums of Lucknow, North India. Over a 2-year period, the ICDS successfully provided regular 6-monthly treatment to 95% of the children targeted, and the treated children gained about an extra kilogram in weight when compared to untreated children in neighbouring slums. These results show that the preschool program in India could provide regular deworming simply and cheaply, and suggest that poor and malnourished preschool children with a heavy worm load could show a substantial gain in weight as a result.

## Introduction

Intestinal nematodes are amongst the most common agents of chronic infection in low income countries [1]. In India, it has been reported that about half the population is infected with round worm (*Ascaris lumbricoides*), whipworm (*Trichuris trichiura*) and/or hookworm (*Necator americanus*, *Ancylostoma duodenale*) [2]. These infections could have significant effects on the development of children [3]. Any effects on physical development would typically be subtle and chronic, manifesting as longstanding anaemia [4]–[6], reduced physical fitness [7], and somewhat constrained growth [8]. There might also be subtle, but important developmental effects on cognition and educational achievement [9]–[11]. Recognition of these potential consequences of infection has led to increasing emphasis on community-based anthelmintic treatment to reduce worm burdens in children irrespective of direct evidence of infection, particularly children of school age [1],[3],[12],[13].

The current focus on schoolchildren reflects three factors: the evidence of an effect on weight and cognition in this age group; the accessibility of school children to treatment delivered through the education infrastructure; and the epidemiological observation that school children tend to harbour the largest worm burdens [12]. Few studies have examined the impact of infection on younger children, partly because the burden of worms and, it has been assumed, disease is light at this early age, and perhaps because of the practical difficulty in reaching the pre-school population. Two recent studies from Africa, however, suggest different conclusions. A study of deworming children during home visits in rural Zanzibar indicated that the youngest children, who are most at risk of growth retardation, showed the greatest increase in growth rate after treatment with anthelmintics [4], while a study of deworming pre-school children during “health days” in Uganda increased growth rate and was cost-effective [14].

The situation in North India suggested that deworming of children younger than school age might be beneficial and practical. In Uttar Pradesh (U.P.) in North India, high levels of worm infection and malnutrition have been observed in pre-school children [15] and our observations indicate that more than a third of pre-school children around Lucknow are infected, predominantly with *Ascaris lumbricoides*. A study of supplementary nutrition suggested some benefit from deworming such children [16]. Furthermore, the State Integrated Child Development Scheme (ICDS) provides a health care infrastructure that aims to reach all children up to 5 or 6 years of age, and thus provides a potentially cost effective mechanism for delivering anthelmintics to these children [17].

This was a cluster-randomized trial, since for a study on this scale randomization by area was the only feasible strategy. The study was conducted using the existing community and health infrastructures to allow the possibility that the interventions could be institutionalised and scaled up with minimum additional inputs.

## Methods

We conducted an open-labelled, cluster-randomized trial within the ICDS infrastructure in urban Lucknow UP with the hypotheses that the ICDS could deliver anthelmintics regularly to pre-school children in the urban slums of Lucknow, and that if they did then this would result in a growth benefit (trial registration number NCT00396500). We obtained approval from the institutional human ethics committee in Lucknow. The primary objective was to assess the impact of 6 monthly deworming on weight and height gain over 2 years in children initially aged 1 to 5 years of age. The protocol for this trial and supporting CONSORT checklist are available as Supporting Information; see Protocol S1 and Checklist S1.

There are 203 designated urban slum areas in urban Lucknow, each of which has about 100 pre-school children and is served by a pre-school centre, known as an anganwadi ( = courtyard) center. An ICDS worker in each area uses the anganwadi center to provide general care to children under 5, including administration of 100,000 units of vitamin A syrup every 6 months. A geographically convenient group of fifty of these slum areas was chosen for the study. All these areas continued their vitamin A supplementation and other usual care during the study period, and 25 were randomly allocated to offer in addition, 400 mg of albendazole (Zentel, SmithKline Beecham) as 10 ml suspension at the same time as the six-monthly vitamin A.

The ICDS workers were provided training regarding: (a) indications of the interventions, precautions during the storage and administration of the interventions, and adverse effects; (b) strategies for community mobilization; (c) record keeping; and (d) procedures for weight and height measurement. Interventions were administered orally in the anganwadi centers by the visiting research team working in collaboration with the local anganwadi center staff. All resident children between 1 and 5 years of age were recruited to the study, and all parents of eligible children provided written consent. None of the parents refused participation, because when, after much discussion, those in such communities chose to cooperate they all became willing to do so for the common good. A research team was trained to measure height to the nearest 0.1 cm, using a stadiometer, and weight to the nearest 0.1 kg, using electronic weighing scales which were calibrated daily. Anthropometry of each child was done at baseline, and then at 6-month intervals for 24 months. Adverse events were not assessed in this trial.

### Sample size

It was expected from cross-sectional surveys that the 2-year weight gain in this poorly nourished population would be only about 3 kg, perhaps with standard deviation (SD) about 1 or 1.5 kg. Exact sample size calculations were not possible because it was not known what fraction f of this variance was due to real differences between different slums. If 25 albendazole and 25 control slum areas are compared, with about 100 children per slum area, then unless f is very small, the standard error of the treatment effect (SE) is approximately 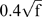 (formally, 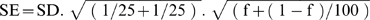; f is sometimes called the intra-cluster correlation coefficient, or ICC). If, for example, f = 1/4 then SE would be about 0.2 kg, so the study would have a reasonable chance of detecting a 2-year weight gain of 0.5 kg.

ICCs for future reference were estimated as the fractional reduction on comparing the residual mean squares after regression on initial age, sex and cluster membership with those after regression only on initial age, sex and treatment allocation.

Random allocation was done by SA, listing the anganwadi centers of each slum area serially in alphabetical order, numbering them from 1 to 50, and then generating a single random number by computer that allocated either all odd or all even numbers to a specific intervention type.

### Analysis

EPI 6.1 statistical software was used to calculate the weight-for-age, height-for-age and weight-for-height z-scores, which compare the study children to the nutritional standards of the World Health Organization. The z-score measures show, in standard deviations, how far a particular measurement departs from these standards. The baseline prevalences of undernutrition (weight-for-age z-score ≤−2), stunting (height-for-age z-score ≤−2) and wasting (weight-for-height z-score ≤−2 ) were calculated for all the enrolled children.

Because randomization was by area, we first assessed the area-specific results, one per area. We then compared these 50 numbers with each other (25 versus 25), and our means and standard errors reflect the variation between these 50 values. For example, height and weight gain in one year and at two years were compared in 25 “usual care” versus 25 “usual care plus albendazole” areas. Reported here are the mean weight and height gains with their standard errors. Weight and height gains were also calculated separately for children who were underweight, stunted and wasted at enrolment in both groups. Although in such analyses area-specific results are affected by regression to the mean, the comparison of 25 versus 25 areas remains unbiased. All p-values refer to two-sided significance tests.

## Results

**Figure 1:**
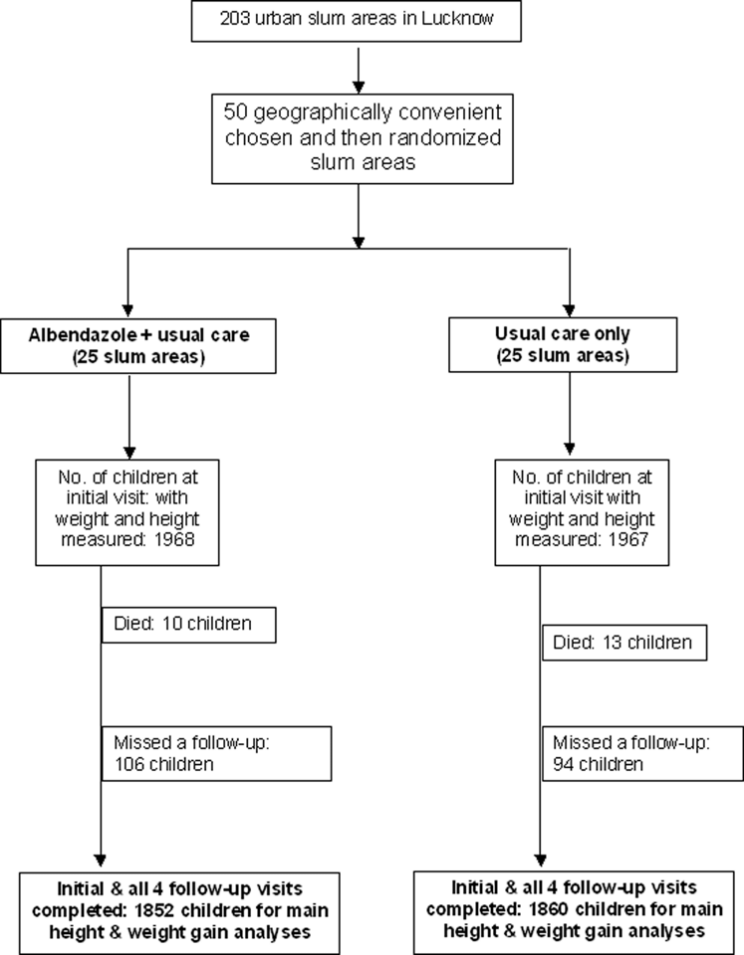
CONSORT Flow Chart

A total of 3935 children, aged 1 to 5 years at entry, 1967 from 25 anganwadi centers allocated “usual care” and 1968 from 25 anganwadi centers allocated “usual care plus albendazole” were enrolled in the study from April to August, 1994 and followed up until December, 1996 (Figure 1). Relative to WHO reference standards, the point prevalence at baseline of underweight was 49% (1924 children), stunting 63% (2471), and wasting 18% (699). The children were all from families with incomes below the national poverty level, and 51% were girls. The children were treated and surveyed 5 times (at 0, 6, 12, 18 and 24 months) and anthropometric data were collected on all 5 occasions from 95% (3712/3912) of the surviving children in each arm of the study. At 24 months, 99% (3855/3912) of the surviving children were measured. There were 23 deaths in 2 years, of which 13 were in the usual care arm and 10 were in the arm receiving usual care plus albendazole.

At the 1 year follow up there was no significant difference in height gain (7.5 [SE 0.3] cm vs. 7.6 [SE 0.2] cm), but the usual care plus albendazole arm had gained significantly more weight (1.57 [SE 0.06] kg vs. 1.93 [SE 0.08] kg, difference 0.36 [SE 0.10] kg, p = 0.0007). At 2 years the albendazole group showed slightly greater height gain (13.4 cm vs. 14.1 cm, difference 0.7 [SE 0.4] cm, p = 0.11) but this difference was still not significant. The usual care plus albendazole arm continued, however, to show a highly significant difference in weight gain (2.8 [SE 0.1] kg vs. 3.8 [SE 0.1] kg, difference 1.0 [SE 0.15] kg, p = 10^−11^), with the dewormed children exhibiting a 35 (SE 5) % higher weight gain. This is equivalent to an additional 1.0 (SE 0.15) kg over 2 years (99% CI: 0.6–1.4 kg). For the 2-year gains in height and weight the intra-cluster correlation coefficients were not zero (0.11 for height gain [95% CI 0.07–0.15]; 0.17 for weight gain [95% CI 0.11–0.23]: see Methods). The SE calculations allow for this, however, as they are based only on the 50 area-specific mean gains.

**Figure 2:**
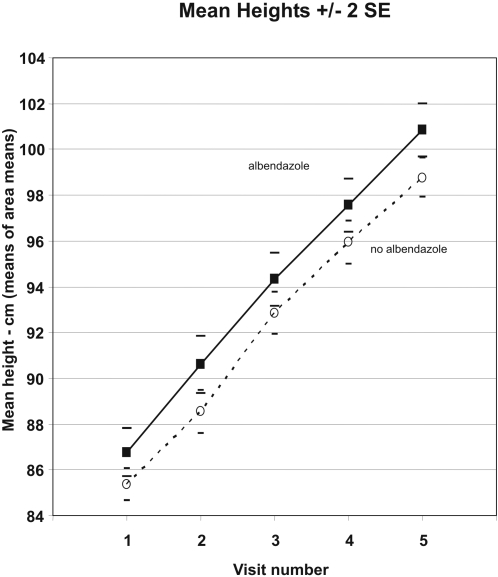
Mean Heights by Visit and Treatment

**Figure 3:**
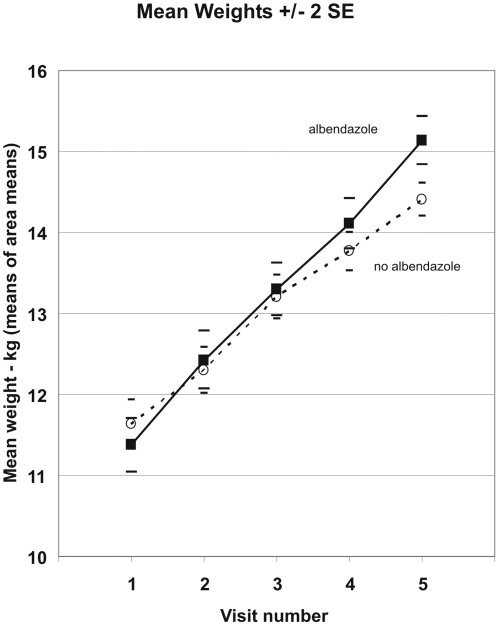
Mean Weights by Visit and Treatment

The mean heights in the two arms at each visit are shown in Figure 2, with all mean values and standard errors calculated only from the 50 (25 vs 25) area-specific mean results. The usual care plus albendazole arm children were, by chance, slightly taller at entry (1.2 [SE 0.6] cm, p = 0.06), and this difference in mean height persisted throughout the study. Figure 3 shows the corresponding results for weight. The usual care plus albendazole arm children had, by chance, slightly lower mean weight at entry (0.3 [SE 0.2] kg, p = 0.19), but as the study progressed this was reversed by the significantly greater weight gain.

The non-significant differences in height and weight at baseline pointed in opposite directions before any treatment had been given. If, as a data-dependent response to these unexpectedly opposite differences, we were to compare baseline weight-for-height, the difference would be significant (p = 0.0013). We regard this, however, after much investigation of the original records, as merely an oddity of the randomization rather than a failure of it, and its effects are appropriately allowed for in the main analysis by our emphasis on mean changes from baseline over the 2-year study period.

We compared the scale of the age-specific weight gain in the usual care arm with the differences in weight between age classes at baseline (Table 1). The observed weight gain in the study was similar to the differences in weight between year classes at the beginning of the study. The growth rate, by both measures, declines with age, as expected. When these data are compared with the weight gains in the usual care plus albendazole arm, there was a significant increase in weight with treatment for all age classes. This indicates some growth benefit even in the younger children who are likely to harbour lower worm burdens.

**Table 1:** Age-specific changes in weight (kg).

Initial age (years)	Initial weight (mean±SE)	Difference in initial weight per year of age	Two-year weight gain during the study (mean±SE)		
			Usual Care	Albendazole	Difference
1–1.99	8.82±0.15		3.24±0.11	4.41±0.14	1.17±0.18
		1.62			
2–2.99	10.44±0.14		2.93±0.11	4.04±0.14	1.11±0.18
		1.30			
3–3.99	11.74±0.14		2.70±0.09	3.83±0.15	1.13±0.17
		1.50			
4–4.99	13.24±0.11		2.45±0.12	3.33±0.13	0.88±0.18

*: Each mean value is an average of one value per slum area, and its standard error (SE) reflects this appropriately.

Table 2 shows that for all children, irrespective of treatment group, the weight gain at 2 years was greater for those children who were malnourished initially. This effect was significant for underweight and wasting, but not for stunting. It is, however, difficult to correct this observation for the effects of measurement error. One way of doing so is to note that the 2-year weight gain is also significantly correlated with the weight at the mid-year. For all nutritional categories, the weight gain was significantly greater for those children who received albendazole in addition to usual care. In the under-nourished categories, the greatest gain in weight was for wasted children and the least for stunted children.

**Table 2:** Weight gain (kg) over two years, usual care versus albendazole, stratified by nutritional status at baseline.

Status at baseline	Usual Care Mean gain±SE	Albendazole Mean gain±SE	*p value* (A vs. UC)
	(UC)	(A)	
a. Underweight*	3.23±0.09 (800)	4.27±0.11 (1016)	*<0.00001*
b. Not underweight	2.42±0.09 (1060)	3.15±0.11 (836)	*<0.00001*
*p value, a vs. b*	*<0.00001*	*<0.00001*	
c. Stunted*	2.80±0.10 (1215)	3.85±0.13 (1122)	*<0.00001*
d. Not stunted	2.76±0.09 (645)	3.60±0.13 (730)	*<0.00001*
*p value, c vs. d*	*0.30*	*0.16*	
e. Wasted*	3.63±0.11 (247)	4.56±0.12 (400)	*<0.00001*
f. Not wasted	2.65±0.08 (1613)	3.56±0.11 (1452)	*<0.00001*
*p value, e vs. f*	*<0.00001*	*<0.00001*	
ALL	2.78±0.09 (1860)	3.76±0.12 (1852)	*<0.00001*

All means and standard errors in the table are derived appropriately from the 50 slum area means, as befits the cluster-randomization scheme. Numbers of children are given in brackets.

*: More than 2 standard deviations below WHO nutritional standards (given sex) for weight-for-age, height-for-age and weight-for-height.

## Discussion

These results suggest that, at the time of the study (mid 1990s), 6 monthly deworming as a part of an ICDS program in these urban slums in north India was associated with substantial weight gain in malnourished pre-school children. The mechanism for this gain was not assessed by the present study design as we have no measure of faecal worm egg counts, but it may reflect a direct effect of deworming on nutrition. The effect may also be indirect since a negative association between ascariasis and vitamin A absorption has been described [18], but a study in an area of low infection prevalence showed a benefit of vitamin A supplementation that was not further enhanced by deworming [19].

A moderate increase in weight but not height appears to be a consistent phenomenon in deworming trials of worm-affected populations. Of five published randomized trials of anthelmintic treatment that showed significantly increased growth in preschool children [4],[14],[20], all reported benefits in ponderal growth, and only one an increase in linear growth. The lack of a significant height gain may reflect an initial increase in tissue mass rather than linear growth for such disadvantaged children; in the children in Lucknow the height gain was greatest for those stunted initially, and least for those wasted (data not shown). The results of the present study are also of similar scale to previous trials where the annual gain is typically in the range 5% to 10% above the control group [4],[14],[20]. The gain observed in the present study was equivalent to an additional 1.0 (SE 0.15) kg over 2 years, and even the lower 99% confidence limit of 0.6 kg represents an additional gain of 0.3 kg per year. Since the size of the populations living in urban slums is increasing world wide, these findings could be of growing relevance to middle and low income countries [21].

In pre-school children there is a direct correlation between weight and relative risk of death. In 8 prospective studies of children 6 to 59 months of age, set in Asia and Africa, the mean relative risk of death from common childhood diseases is 8.4 (SE 2.1) with severe, 4.6 (SE 0.9) with moderate and 2.5 (SE 0.3) for mild malnutrition (where severe malnutrition is weight-for-age z-score <−3, moderate is between −3 and −2 and mild is between −2 and −1) [22] . However, 80% of deaths in children under five years of age occur in those that are only mildly malnourished [22] . In India in particular a high proportion of mortality in children aged 1–5 years old may be attributed to the potentiating effects of malnutrition on infectious disease [22]. In the present study the scale of weight gain that can be achieved in poor urban settings in India might be sufficient to contribute to reduced mortality, although a very large study would be needed to demonstrate this directly.

This study suggests that deworming tablets which costs less than US$ 0.10 per annum, and can be delivered through the existing ICDS infrastructure, could contribute to improved child growth and, perhaps, survival in urban north India.

## Supporting Information

Protocol S1 Prepilot Proposal
(0.04 MB DOC)

Checklist S1 CONSORT Checklist
(0.04 MB DOC)
