# Supplementary material for: Effects of Deworming on Malnourished Preschool Children in India: An Open-Labelled, Cluster-Randomized Trial
Source: PLoS Negl Trop Dis. 2008 Apr 16;2(4):e223. doi: 10.1371/journal.pntd.0000223 (PMC2291568; doi:10.1371/journal.pntd.0000223)
Supplement: Protocol S1 — Prepilot Proposal (0.04 MB DOC) [file pntd.0000223.s001.doc]

Title: To assess the effectiveness of six-monthly deworming with Vitamin A administration on growth in 1-5 year old children in the urban slums of Lucknow

Principal Investigator: Shally Awasthi,

Department of Pediatrics

King George's Medical College, Lucknow

Co-investigators: Dr. Richard Peto, CTSU, Oxford

Dr. DAP Bundy, Oxford University

Dr. VK Pande, KGMC

Prof Robert Fletcher, Harvard Medical School

***Background***

It has been estimated that more than one third the world's population is infected with one or several species of parasitic worms and that in the Indian subcontinent, there were 160 million cases of ascariasis and hookworm infestation each. The consequences of such infestations are malnutrition and anemia. Infestation begins from the time children are weaned, around the age of six months, and by the time they reach the school going age helminths are the leading cause of disability. The critical period for malnutrition is between 6 months to 24 months of age, which also coincides with the period where worm infestations begin. The most appropriate period, then, to tackle malnutrition, and thereby reduce malnutrition related mortality, is in the preschool age group. In this age, it has been estimated that 42-57% of deaths are due to malnutrition's potential effects on infectious diseases, of which 76-89% is attributed to mild-to-moderate malnutrition.

A number of studies have identified an improvement in nutritional status following deworming treatment, but it is evident that findings vary. Some trials have reported significant improvements in growth, whereas others have failed to detect a change.

We, therefore, conducted a randomized trial to study of the effects of albendazole on growth in preschool children . Our study hypothsis was that albendazole administration six monthly, as a single 400 mg dose in syrup, by the existing health care delivery system would be a practicable way to achieve mass deworming of preschool children and this might result in an improvement in weight gain of preschool children.

# Review of Literature

**Parasitic worms** (helminths) are a common cause of chronic infection in humans. This review concerns the effects of deworming for soil-transmitted worms only, and does not cover schistosomiasis. World Bank estimates in 1993 ranked soil-transmitted worms first in terms of global burden of disease in the 5-14 year age group (World Bank 1993). Global estimates suggest that more than a quarter of the world’s population are infected with one or more of the most common soil-transmitted intestinal worms (nematode geohelminths) (Chan 1997). These include roundworms (Ascaris lumbricoides), hookworms (Necator americanus and Ancylostoma duodenale) and whipworms (Trichuris trichura). Most infected people harbour multiple species. Infections are widely distributed in tropical and sub-tropical areas. The burden of disease falls disproportionately on the poor, where inadequate sanitation, overcrowded conditions, low levels of education and lack of access to health make them particularly susceptible.

The distribution of worms in mammalian hosts is highly skewed. In a population certain people harbour particularly high worm burdens, whereas others carry just a few parasites. Studies have also shown a correlation between individual pre-treatment worm intensity and that attained on re-infection after treatment. This may reflect differences in exposure and susceptibility to infection, and variations in worm survival. Children are more at risk of high worm burdens, with peak intensities at 5-10 years for roundworms and whipworms. Hookworm burden peaks later.

Roundworms obtain their nutrition from gastrointestinal contents. There is an association with malnutrition, possibly mediated through impaired fat digestion, reduced vitamin absorption (particularly vitamin A) and temporary lactose intolerance (WHO 2002). Whipworm infection has also been associated with malnutrition, the precise mechanism for this being unclear (Cappello 2004). Suggested mechanisms for worm effects on growth include appetite suppression, increased nutrient loss, and decreased nutrient absorption and utilisation (Stephenson 2000, deSilva 2003).

**Albendazole:** The drugs that have been used in the current decade for mass deworming are mebendazole and albendazole (1,16). Albendazole is a benzimidazole derivative and has a broad spectrum antihelminthic effect (16). We have used albendazole because it is usually administered in a single dose. It is so safe that it can be used by non-mediacl personnel within a program. The cure rates of 400 mg of the drug in subjects of all ages and those between 2 to 14 years of age is 100% for ascariasis (17,18) and 63 to 84% for hookworms, the two different cure rates are based on the technique for identification of the hookworms (18). The drug has been used in children less than 2 years of age without any significant side effects (19).

Albendazole is being used for school based deworming programs where the measure of effectiveness is gain in weight. With albendazole, we have demonstrated weight gain even in the preschool children.

## Design

### Study Hypothesis

. Our study hypothsis is that albendazole administration six monthly, as a single 400 mg dose in syrup, by the existing health care delivery system would be a practicable way to achieve mass deworming of preschool children and this might result in an improvement in weight gain of preschool children.

### Objectives

Primary objective:

1. To assess the impact of 6 monthly deworming on weight and height gain at the end of 2 years in children aged 1 to 5 years of age

### Setting

The study will be conducted in urban slums of Lucknow, a heavily populated area of North Central India. There is one slum, or *'anganwadi'* for a population of approximately 1000 economically disadvantaged persons. The criteria for selecting an area for an urban slum are that it does not have the basic amenities of sanitation and water supply and the income of its residents is below the national poverty level. To ensure primary health care of children under five years of age, all the urban slums in Lucknow are covered by the national Integrated Child Development Scheme (ICDS). Through the ICDS, there is one informal health worker, the *'anganwadi'* worker per slum and the entire slum population is registered with her. There are 203 slums within urban Lucknow. These will be listed alphabetically and given a serial number. After generating a random digit table with 55 number, the first slum will be randomized to either one intervention type by toss of coin, and then every alternate slum having the same serial number as that in the random digit table will receive this intervention, the rest the other. Thus, 50 slums will be randomly allocated, 25 to each of the two arms of the trial.

### Design

This study will be a cluster randomized open labeled trial within the existing health care infrastructure. It will be conducted after clearance from the local institutional ethics committee.

### Intervention

One group will received usual health care by the existing health care staff, which included six monthly administration of Vitamin A concentrate. The other group will receive, in addition, 400 mg of albendazole (Zentel, Smith Kline & Beecham) in 10 ml syrup form. Five such doses will be given given at six monthly intervals for 2 years.

### Unit of randomization

Randomization will be by the urban slums.

### Selection of Subjects

All the children residing in the slums and between 1 through 5 years of age will be invited for participation in the study, after obtaining written consent from the parents. The date of birth of children was noted from the register maintained by the slum health workers.

### Variables

The main outcome measures are weight and height. The project workers will measure these on the day of the visit, once every six months. To ascertain age of the hild, date of birth will be abstracted from the register of the '*anganwadi'* worker. For those who cannot be contacted at the time of the visit but were not reported as dead, a second visit will be made to ascertain the reason for loss to follow-up. Parents of dead children will be contacted to ascertain the cause of death.

The weight of the child will be measured in kilograms to the nearest 100 grams and the standing height was measured in centimeters to the nearest millimeter. It will not be possible to blind the data collectors or the parents to the intervention type. However, the data collectors did not have information of the previous weight or height of the children. Approximately 10% of the data will be validated by different project workers within 1 week. The validators will be blind to all the anthropometric data on the subjects.

### Compliance

The project staff themselves will administer the intervention. Independent data collectors will validate this in 10% children within 15 days. Empty bottles of intervention will be retained and counted. The numbers of empty bottles and the doses within them will be compared with the doses administered.

### Sample Size and Data Analyses

Sample Size: Sample size was calculated for a continuous outcome. For a standardized effect size of 0.1, with a power of 80% and an alpha level of 0.05, using a 2-tailed t test, taking into account design effect, about 2000 children will be included in each arm.

Data Analysis: EPI6.1 statistical software will be used to calculate at baseline, weight for age (WA), height for age (HA) and weight for height (WH) z scores, when compared to the nutritional standards of the World Health Organization. Baseline distribution of undernutrition (weight for age (WAZ) z score < - 2 standard deviations (S.D.)), stunting (height for age (HAZ) z score < - 2 S.D.) and wasting (weight for height (WHZ) z score < - 2 S.D.) will be calculated for all the enrolled children. Weight by age will be calculated for all the enrolled children using the 1994 data of the first survey. Univariate analyses will be done to compare age, sex and history of passage of worms and weight and height in 25 control areas and 25 albendazole areas using the chi square test for categorical and student's t test for continuous variable.

Because randomization will be by area, we will assess the area specific weight gain and height gain in each group. We will report area specific averages, taking one result per area. Height and weight gain in one year and two years will be compared in 25 control versus 25 albendazole areas.

In subgroup analysis, weight and height gains will be calculated for children who are underweight, stunted and wasted at enrollment in the control and ABZ groups. We will also compare 2-year changes in the proportion of underweight, stunted and wasted in the two groups.

#### References

1. Bundy DAP, Wong MS, Lewis LL, Horton J. Control of gastrointestinal helminths by age-group targetted chemotherapy delivered through schools. Trans Royal Soc Trop Med Hygiene, 1989; 8: 115 - 120.
2. deSilva DG, Hettiarachchi SP, Fonseka PM. Albendazole in treatment of geohelminth infections in children. Ceylon Med Journ, 1989; 34: 185 - 189.
3. Eggler RJ, Hofhuis EWH, Bloen MW, Chusilp K, Wedel M, Intrakhao C et al. Association between intestinal parasitosies and nutritional status in 3-8 years old in north-east Thailand. Trop Geogr. Med, 1990; 42: 312 -323.
4. Grant JP. The State of World's Children, 1994 (UNICEF). Oxford University Press.
5. Jamison DT and Leslie J. Health and nutritional considerations in educational planning: the cost and effectiveness of school based intervantions. Food and Nutrition Bulletin, 1990; 12: 204 - 214.
6. Kazura Jw, Mahm AAF. Intestinal Nematodes. In Nelson's Textbook of Pediatrics. Eds. Behrman RE, Kliegman RM, Nelson WE, Vaughan VC. 14 th Edition, WB Saunders Co., Philadelphia. 1992, pp 896 - 899.
7. Kvalsvig JD, Cooppan RM, Connolly KL. The effects of parasite infections on cognitive processes in children. Ann Trop Med Parasitol, 1991; 85: 551 - 568.
8. National Child Survival an Safe Motherhood Program. MCH Division, Department of Family Welfare, Government Of India, January 1994. Acute Respiratory Infections, pp. 50 - 56.
9. National Child Survival an Safe Motherhood Program. MCH Division, Department of Family Welfare, Government Of India, January 1994. Management of diarrheal diseases, pp. 79 - 85.
10. Nokes C, Grantham McGregor SM, saywer AW, Cooper et al. Parasitic helminthic infection and cognitive function in school children. Proc R Soc Lond B, 1992; 247: 77 - 81.
11. Pamba HO, Burbo NO, Chunge CN, Estambale BB. A study of the efficacy and safety of albendazole (Zentel) in the treatment of intestinal helminthiasis in Kenyan children less than 2 years of age. east Afr Med Journ, 1989; 66: 197 - 202.
12. Pelletier Dl, Frongillo EA Jr, Schroeder DG, Habicht JP. A methodology for estimating the contribution of malnutrition to child mortality in developing countries. J Nutr 1994 (10 Suppl); 2106S - 2122S. (meta-analysis of eight prospective studies for association of weight & mortality)
13. Prescott NM, Jancloes MF. The analysis and assessment of health programs. Social Science and Medicine, 1984; 19: 1057 - 1060.
14. Raccurt CP, Lambert MT, Bouloumie J, Ripert C> Evaluation of the treatment of intestinal helminthiasis with albendazole in Djong (North Cameroon). Trop Med Parasitology, 1990; 41: 46 - 48.
15. Sokhey J, Kim Farley RJ, Bhargav I. Case definition in surveillance of vaccine preventable disease. Indian Pediatrics, 1988; 25: 600 - 603.
16. Stephson LS, Latham MC, Adams EJ, Kinoti SN, Pertet A. Physical fitness, growth and appetite of Kenyan school boys with hookworm, trichuris trichura and ascaris lumbricoids infections are improved four months after a single dose of albendazole. Journ Nutrition, 1993; 123: 1036 - 1046.
17. Stephson LS, Latham MC, Adams EJ, Kinoti SN, Pertet A. Weight gain of Kenyan school children infected with hookworm, trichuris trichura and ascaris lumbricoids improving following once or twice yearly treatment with albendazole. Journ Nutrition, 1993; 123: 656 - 665.
18. Warren KS, Bundy DAP, Anderson RM, Davis AR, Henderson DA, Jamison DT, Prescott N, Senft A. Helminthic Infections. In Disease Control Priorities in Developing Countries Eds. Jamison DT, Mosley WH, Measham AR, Bobadilla JL. Oxford University Press, New York, 1993, pp.131 - 160.
19. Webster LT. Drugs used in chemotherapy of helminthiasis. In Pharmacological Basis of Therapeutics. Ed A. Goodman Gilman, TW Rad, AS Nies, P Taylor, pp. 959 - 977.
20. WHO Expert Committee. Public health significance of intestinal parasitic infections. Bull World Health Organization, 1987; 65: 575 - 588.
21. World Developing Report 1993: Investing in Health.New York University Press, pp. 204-222.
