# Supplementary material for: Effects of Deworming on Malnourished Preschool Children in India: An Open-Labelled, Cluster-Randomized Trial
Source: PLoS Negl Trop Dis. 2008 Apr 16;2(4):e223. doi: 10.1371/journal.pntd.0000223 (PMC2291568; doi:10.1371/journal.pntd.0000223)
Supplement: Checklist S1 — CONSORT Checklist (0.04 MB DOC) [file pntd.0000223.s002.doc]

Checklist of items to include when reporting a cluster randomised trial (adaptations from standard guidelines in italic)

| PAPER SECTION And topic | Item | Description | Reported on  Page # |
| --- | --- | --- | --- |
| *TITLE & ABSTRACT* | 1 | How participants were allocated to interventions (*e.g*., "random allocation", "randomized", or "randomly assigned"). *specifying that allocation was based on clusters* | 1 |
| *INTRODUCTION* Background | 2 | Scientific background and explanation of rationale. *including the rationale for using a cluster design* | 3 (para 3) -4 (para 1,2) |
| *METHODS* Participants | 3 | Eligibility criteria for participants *and clusters* and the settings and locations where the data were collected. | 4 (para 3,4) |
| Interventions | 4 | Precise details of the interventions intended for each group , *whether they pertain to the individual level, the cluster level, or both*, and how and when they were actually administered. | 4 (para 4) |
| Objectives | 5 | Specific objectives and hypotheses *and whether they pertain to the individual level, the cluster level, or both* | 4 (para 3) |
| Outcomes | 6 | Clearly defined primary and secondary outcome measures *whether they pertain to the individual level, the cluster level, or both*, and, when applicable, any methods used to enhance the quality of measurements (*e.g.*, multiple observations, training of assessors). | 5 (para 2) |
| Sample size | 7 | How *total* sample size was determined *(including method of calculation, number of clusters, cluster size, a coefficient of intracluster correlation (ICC or k), and an indication of its uncertainty)* and, when applicable, explanation of any interim analyses and stopping rules. | 5 (para 2) |
| Randomization -- Sequence generation | 8 | Method used to generate the random allocation sequence, including details of any restrictions (*e.g*., blocking, stratification, *matching*) | 5 (para 2) |
| Randomization -- Allocation concealment | 9 | Method used to implement the random allocation sequence *specifying that allocation was based on clusters rather than individuals and clarifying*, clarifying whether the sequence was concealed until interventions were assigned. | 5 (para 2) |
| Randomization -- Implementation | 10 | Who generated the allocation sequence, who enrolled participants, and who assigned participants to their groups? | 5 (para 2) |
| Blinding (masking) | 11 | Whether or not participants, those administering the interventions, and those assessing the outcomes were blinded to group assignment. When relevant, how the success of blinding was evaluated. | 5 (para 2) |
| Statistical methods | 12 | Statistical methods used to compare groups for primary outcome(s) *indicating how clustering was taken into account*, methods for additional analyses, such as subgroup analyses and adjusted analyses | 5 (para 3), 6 (para 1) |
| RESULTS  Participant flow | 13 | Flow of *clusters and* individual participants through each stage (a diagram is strongly recommended). Specifically, for each group report the numbers of *clusters and* participants randomly assigned, receiving intended treatment, completing the study protocol, and analysed for the primary outcome. Describe protocol deviations from study as planned, together with reasons | 6 (para 2), 12 |
| Recruitment | 14 | Dates defining the periods of recruitment and follow-up. | 6 (para 2) |
| Baseline data | 15 | Baseline demographic and clinical characteristics of each group *for the individual and cluster levels as applicable*. | 6 (para 2) |
| Numbers analyzed | 16 | Number of *clusters and* participants (denominator) in each group included in each analysis and whether the analysis was by "intention-to-treat". State the results in absolute numbers when feasible (*e.g*., 10/20, not 50%). | 6 (para 2), 7 (para 1&2) |
| Outcomes and estimation | 17 | For each primary and secondary outcome, a summary of results for each group *for the individual or cluster level as applicable*, and the estimated effect size and its precision (eg 95% confidence interval) *and a coefficient of intracluster correlation (ICC or k) for each primary outcome.* | 6 (para 3), 16 |
| Ancillary analyses | 18 | Address multiplicity by reporting any other analyses performed, including subgroup analyses and adjusted analyses, indicating those pre-specified and those exploratory. | 7 (para 3 & 4), 16 |
| Adverse events | 19 | All important adverse events or side effects in each intervention group. | not assessed |
| DISCUSSION Interpretation | 20 | Interpretation of the results, taking into account study hypotheses, sources of potential bias or imprecision and the dangers associated with multiplicity of analyses and outcomes. | 7 (para 2-4), 8 (para 1) |
| Generalizability | 21 | Generalisability (external validity) *to individuals and/or clusters (as relevant)* of the trial findings | 9 (para 2) |
| Overall evidence | 22 | General interpretation of the results in the context of current evidence. | 9 (para 3) |
